# Renal tissue alterations were size-dependent with smaller ones induced more effects and related with time exposure of gold nanoparticles

**DOI:** 10.1186/1476-511X-10-163

**Published:** 2011-09-21

**Authors:** Mohamed Anwar K Abdelhalim, Bashir M Jarrar

**Affiliations:** 1 Department of Physics and Astronomy, College of Science, King Saud University, Saudi Arabia; 2 College of Applied Medical Sciences, Al-Jouf University, P.O. Box (2014), Skaka - Al-Jouf, Saudi Arabia

**Keywords:** gold nanoparticles, renal tissue, histological alterations, hydropic degeneration, nanotoxicity

## Abstract

**Background:**

Gold nanoparticles (GNPs) have important application for cell labeling and imaging, drug delivery, diagnostic and therapeutic purposes mainly in cancer. Nanoparticles (NPs) are being increasingly exploited for medical applications. The aim of the present study was to investigate the particle-size and period effects of administration of GNPs on the renal tissue in an attempt to address their potential toxicity.

**Methods:**

A total of 70 healthy male Wistar-Kyoto rats were exposed to GNPs received 50 or 100 μl of GNPs infusion of size (10, 20 and 50 nm for 3 or 7 days) to investigate particle-size effect of GNPs on the renal tissue. Animals were randomly divided into groups, 6 GNPs-treated rats groups and one control group. Groups 1, 2 and 3 received infusion of 50 μl GNPs of size 10 nm (3 or 7 days), size 20 nm (3 or 7 days) and 50 nm (3 or 7 days), respectively; while groups 4, 5 and 6 received infusion of 100 μl GNPs of size 10 nm, size 20 nm and 50 nm, respectively. Stained sections of control and treated rats kidneys were examined for renal tissue alterations induced by GNPs.

**Results:**

In comparison with respective control rats, exposure to GNPs doses has produced the following renal tubular alterations: cloudy swelling, vacuolar degeneration, hyaline droplets and casts, anisokaryosis, karopyknosis, karyorrhexis and karyolysis. The glomeruli showed moderate congestion with no hypercelluraity, mesangial proliferation or basement membrane thickening. The histological alterations were mainly seen in the cortex and the proximal renal convoluted tubules were more affected than the distal ones.

**Conclusions:**

The induced histological alterations might be an indication of injured renal tubules due to GNPs toxicity that became unable to deal with the accumulated residues resulting from metabolic and structural disturbances caused by these NPs. The findings may suggest that GNPs interact with proteins and enzymes of the renal tissue interfering with the antioxidant defense mechanism and leading to reactive oxygen species (ROS) generation which in turn may induce stress in the renal cells to undergo atrophy and necrosis. The produced alterations were size-dependent with smaller ones induced more affects and related with time exposure of GNPs.

## Introduction

Nanoparticles are an intermediate state of matter somewhere between bulk and molecular level. These particles have important application for cell labeling and imaging, drug delivery, biological sensors, diagnostic and therapeutic purposes mainly in cancer and photodynamic therapy [1-6]. Studies revealed that the NPs were rapidly taken into the system with the highest accumulation in the liver, spleen, lungs, aorta, esophagus and olfactory bulb [7]. Moreover, particles of nano-dimension are believed to be more biologically reactive than their bulk counter parts due to their small size and larger surface area to volume ratio [7,8].

Gold in its bulk form has long been considered an inert, noble metal with some therapeutic and even medicinal value hence GNPs are thought also to be relatively non-cytotoxic [9]. Yet there are differing reports of the extent of the toxic nature of these particles owing to their different modifications, surface functional attachments, shape and size [10,11]. Moreover, the metallic nature of the metal derived NPs and the presence of transition metals encourages the production of reactive oxygen species (ROS) leading to oxidative stress [12,13].

Although some scientists consider NPs as nontoxic, there are other studies reporting the toxic effects of NPs [14-16]. While some NPs may appear to be nontoxic, other cellular mechanisms such as cell signaling and other normal cellular functions may be disrupted and are currently undergoing further investigation [17,18]. The toxicity of NPs is being addressed by a number of standardized approaches with in vitro, in vivo as well as detailed genomic or biodistribution studies [18]. In addition, it has been shown that NPs may produce in vitro toxicity in some cell-based assays, but not in others. This may be a result of interference with the chemical probes, differences in the innate response of particular cell types, or other factors, a point to be considered when GNPs are used as carriers for the delivery of drugs and in gene therapy [19,20].

While nanotoxicity research is now gaining attention, little is paid to the effect of GNPs size mainly to their distribution and alterations in the tissue [21,22]. The histological and the histochemical alterations in the renal tissues due to GNPs have not well documented and have not yet been identified. In the present study, an attempt has been made to address the possible histological alterations in the renal tissues following exposure to GNPs and whether the potential nanotoxicity is related to the size of these particles and the time of exposure.

## Materials and Methods

A total of 70 healthy male Wistar-Kyoto rats obtained from the Laboratory Animal Center (College of Pharmacy, King Saud University, Saudi Arabia). The rats nearly of the same age (12 weeks old) and weighing 220-240 gm of King Saud University colony were used. Animals were randomly divided into groups, 6 GNPs-treated rats groups and one control group. Following a period of stabilization (7 days), 10, 20 and 50 nm GNPs were administered intraperitonealy at the rate for 3 or 7 days as follows: Group 1: received infusion of 50 μl GNPs of size 10 nm for 3 or 7 days (n = 10); Group 2: received infusion of 50 μl GNPs of size 20 nm for 3 or 7 days (n = 10); Group 3: received infusion of 50 μl GNPs of size 50 nm for 3 or 7 days (n = 10); Group 4: received infusion of 100 μl GNPs of size 10 nm for 3 or 7 days; (n = 10); Group 5: received infusion of 100 μl GNPs of size 20 nm for 3 or 7 days (n = 10); Group 6: received infusion of 100 μl GNPs of size 50 nm for 3 or 7 days; (n = 10); Control group: received no gold nanoparticles (n = 10).

The rats were maintained on standard laboratory rodent diet pellets and were housed in humidity and temperature-controlled ventilated cages on a 12 h day/night cycle. Two animals from each group were killed by dislocation of the neck at intervals of 3 and 7 days of treatment with GNPs. All experiments were conducted in accordance with the guidelines approved by King Saud University Local Animal Care and Use Committee.

Fresh portions of both kidneys from each rat were cut rapidly, fixed in neutral buffered formalin (10%), then dehydrated, with grades of ethanol (70, 80, 90, 95 and 100%). Dehydration was then followed by clearing the samples in 2 changes of xylene. Samples were then impregnated with 2 changes of molten paraffin wax, then embedded and blocked out. Paraffin sections (4-5 um) were stained with hematoxylin and eosin stain according to Pearse [23]. Stained sections of control and treated rats were examined for alterations in renal tissue.

## Results and Discussions

No mortality occurred in any of the experimental groups of the present investigation, and no alterations were observed in the appearance and behavior of GNPs treated rats in comparison with the control ones. In comparison with the control group, the following histological alterations were detected in the renal tissue of GNPs treated rats:

### Glomerular alterations

**Figure 1:**
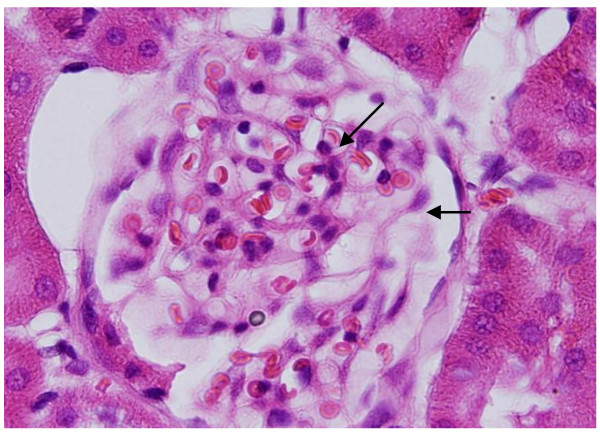
**GNPs-treated rat received 50 μl of 10 nm particles for 3 days demonstrating glomerular congestion (arrow)**.

GNPs produced occasional glomerular congestion in the rats exposed to 10 nm or 20 nm particles for 7 days but not in the glomeruli of the rats exposed to 50 nm **(**Figure 1**)**. No hypercellularity, mesangial proliferation or glomerular basement membranes thickening were detected in the glomeruli of all GNPs treated rats. Occasional dilatation of glomerular tuft blood capillaries was observed. These little alteration showed by the glomeruli might be due to the glomerular basement membrane which forms a barrier that prevents nanoparticles accumulation. Terentyuk et al., 2009 [24] reported proliferation of epithelial cells of Bowman's capsule by GNPs where 15 nm particles showed more effect than larger ones.

### Tubular alterations

The following tubular alterations due to GNPs intoxication appeared in the renal tissue of the treated rats.

**Figure 2:**
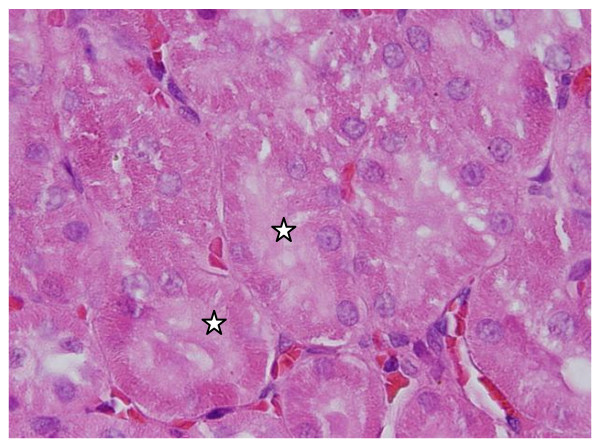
**GNPs-treated rat received 100 μl of 10 nm particles for 7 days demonstrating cloudy swelling of the renal cells (stars)**.

**Cloudy swelling: **renal tubules epithelial lining exhibited cloudy swelling with pale cytoplasm and poorly delineated and displaced nuclei in all GNPs treated rats. This alteration was more prominent in the proximal convoluted tubules than the distal ones and with more swelling with 100 μl dose than 50 μl one and with 10 nm and 20 nm size particles than the larger ones **(**Figure 2**)**. Cytoplasmic swelling might be exhibited as a result of disturbances of membranes function that lead to massive influx of water and Na^+ ^due to GNPS effects. This alteration might be accompanied by leakage of liposome hydrolytic enzymes that lead to cytoplasmic degeneration and macromolecular crowding [25].

**Figure 3:**
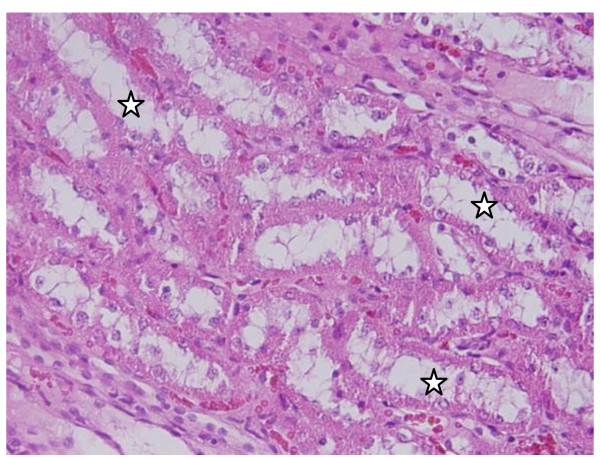
**GNPs-treated rat received 50 μl of 10 nm particles for 3 days showing vacuolar degeneration (stars)**.

**Figure 4:**
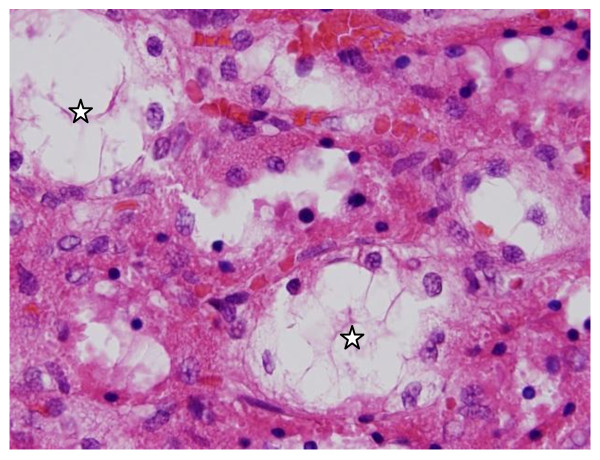
**GNPs-treated rat received 100 μl of 20 nm particles for 7 days demonstrating vacuolar degeneration (stars)**.

**Vacuolar degeneration: **vacuolization of the renal cells was seen and increased in severity in the renal tubules of rats received 100 μl of 10 or 20 nm GNPs with less or no vacuolar degeneration with 50 nm particles. More vacuolar degeneration was observed in the renal cells of rats exposed to 7 days than ones exposed to 3 days (Figures 3 and 4). The proximal renal tubules were more affected than the distal ones that became flattened. This could be due to the fact that the proximal convoluted tubules are the primary sites of reabsorption and active transport leading to higher concentration of the nanoparticles specially the smaller ones in the epithelial lining of these tubules. Vacuolated degeneration is a result of ion and fluid homestasis that lead to an increase of intracellular water [26]. The vacuolated swelling of the cytoplasm of the renal cells of the GNPs treated rats might indicate acute and subacute renal injury induced by these NPs.

**Figure 5:**
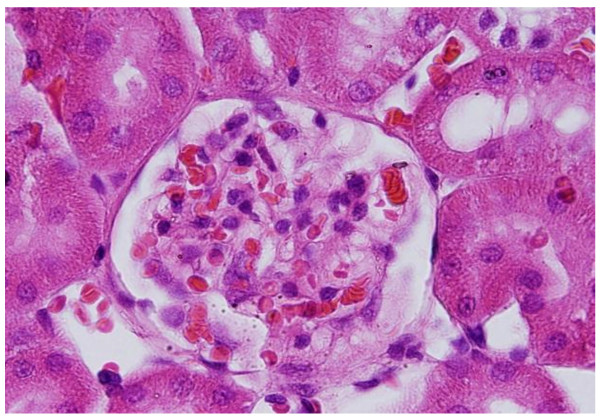
**GNPs-treated rat received 50 μl of 10 nm particles for 3 days demonstrating hyaline droplets in the cytoplasm of the renal cells (arrows)**.

**Figure 6:**
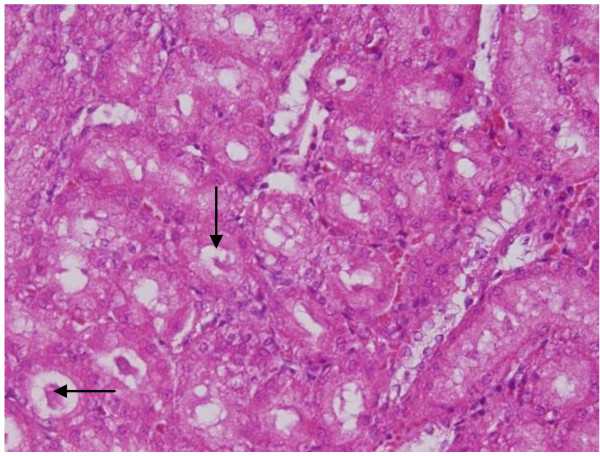
**GNPs-treated rat received 100 μl of 20 nm particles for 7 days demonstrating hyaline casts in some distal tubules (arrows)**.

**Hyaline droplets: **hyaline droplets were detected in the renal epithelium of rats received 100 μl of 10 or 20 nm GNPs **(**Figure 5**)**. Droplets appearance is associated with protein metabolism disturbances. This alteration was not seen in the renal tissue of rats exposed to 50 nm particles. Hyaline casts were also seen in the lumen of some distal convoluted renal tubules **(**Figure 6**)**.

**Figure 7:**
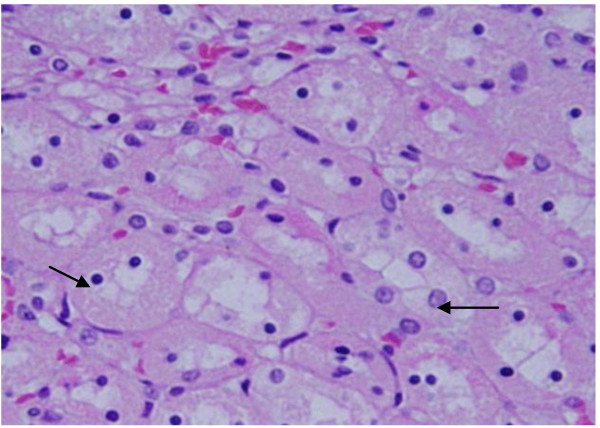
**GNPs-treated rat received 10 μl of 20 nm particles for 7 days demonstrating anisokaryosis (arrows)**.

**Anisokaryosis**: variable nuclei sizes were observed in some renal cells. This change became apparent after 7 days of 50 nm GNPs administration **(**Figure 7**)**. Some studies indicate that nuclear polymorphism is seen in dysplasia and carcinomatous lesion [27].

**Figure 8:**
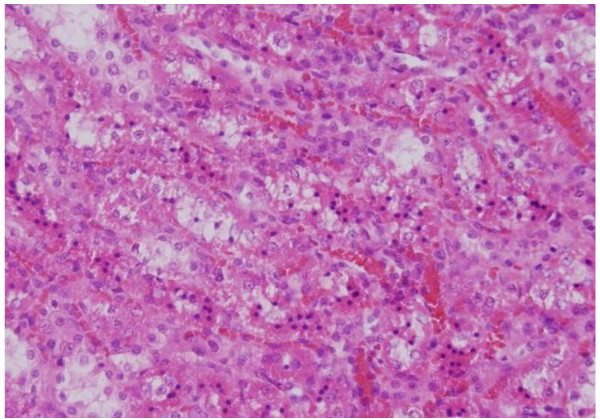
**GNPs-treated rat received 100 μl of 50 nm particles for 7 days demonstrating karyopyknosis**.

**Nuclear pyknosis: **sections of GNPs-treated kidneys developed pyknosis in some epithelium lining cells of the proximal tubules with lesser extent in the distal ones. This alteration was seen in all GNPs-treated rats with 10 and 20 nm particles **(**Figure 8**)**. Pyknotic nuclei exhibited clumping and condensation of the chromatin materials in the periphery of the nuclei together with irregularity nuclear membranes. Karyopyknosis is an irreversible condensation of chromatin in the nucleus of a cell undergoing necrosis or apoptosis [28].

**Figure 9:**
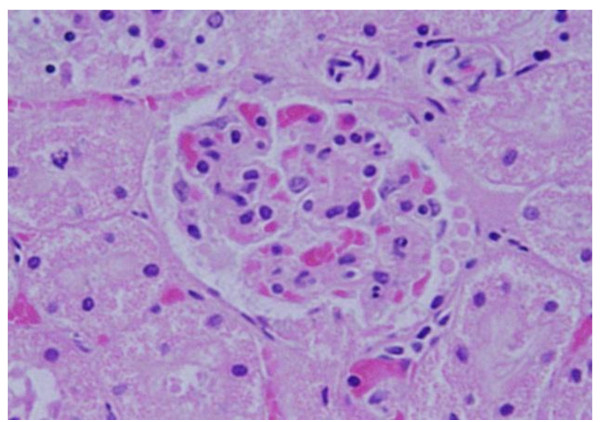
**GNPs-treated rat received 100 μl of 20 nm particles for 7 days demonstrating karyorrhexis lining epithelia of the proximal convoluted tubule (arrows)**.

**Karyorrhexis**: some renal cells of rats received 10 and 20 nm GNPs showed nucleoli disappearance **(**Figure 9**)**. This nuclear damage was more prominent after

7 days of exposure to NPs. Karyorrhexis is a sort of destructive fragmentation of the nucleus proceeded by pyknosis and is followed by karyolysis [29].

**Figure 10:**
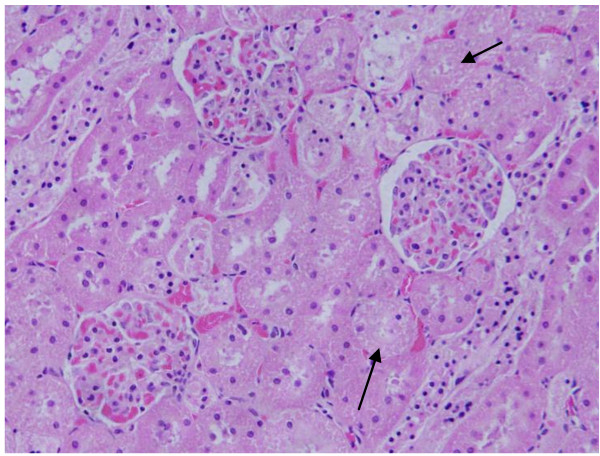
**GNPs-treated rat received 50 μl of 20 nm particles for 7 days demonstrating karyolysis in the renal cells (arrows)**.

**Karyolysis**: this alteration appeared mainly in the kidney of GNPs-treated rats exposed to 100 μl of 20 nm size particles (Figure 10). Karyolysis is the complete dissolution of the chromatin matter of a dying cell [30].

**Figure 11:**
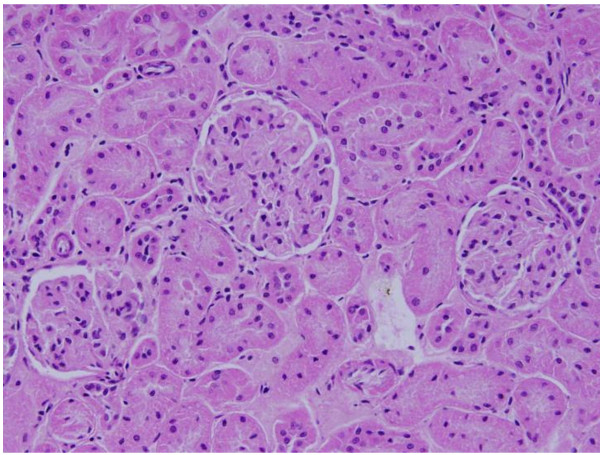
**GNPs-treated rat received 50 μl of 50 nm particles for 3 days demonstrating normal glomerular structure**.

**Figure 12:**
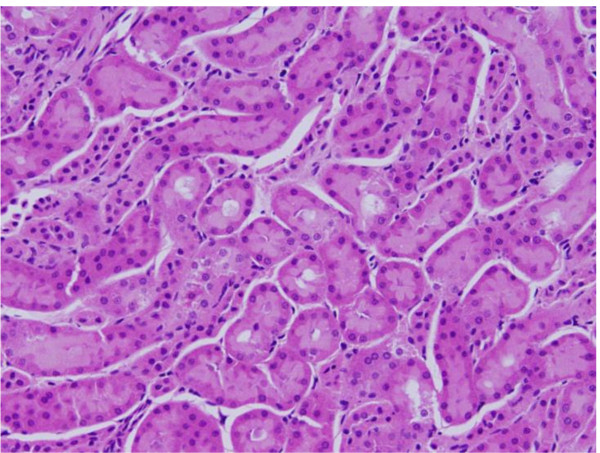
**GNPs-treated rat received 100 μl of 50 nm particles for 7 days demonstrating normal renal tubules**.

The renal tissue of the rats received 50 or 100 μl of 50 nm Au NPs for 3 or 7 days showed little or no alterations while none of the above alterations were observed in the renal tissue of any member of the control group (Figures 11&12).

## Conclusions

Histological alterations by GNPs exposure as shown in the results of the present work could be an indication of injured renal tissue due to GNPs toxicity that become unable to deal with the accumulated residues resulting from metabolic and structural disturbances caused by these particles. One might conclude that these alterations are size-dependent with smaller ones induced more damage to renal tissue with relation to the time exposure of GNPs. This might be due to earlier accumulation of the larger NPs in the tissue while the smaller ones stay much longer in the blood stream due to recirculation.

The appearance of renal cells cytoplasmic degeneration and nuclear destruction may suggest that GNPs interact with proteins and enzymes of the renal tissue interfering with the antioxidant defense mechanism and leading to generation and accumulation of the reactive oxygen species (ROS) which in turn may inflammatory response and mitochondrial destruction inducing stress in the renal cells to undergo atrophy and necrosis and programmed cell death.

More histomorphologcal, histochemical and ultrastrucural investigations are needed to correlate the biomedical application of GNPs with the potential threat of their therapeutic and diagnostic use in correlation with the size, chemical composition, surface charge, solubility and surface structure of these particles.

## Competing interests

The authors declare that they have no competing interests.

## Authors' contributions

MAKA and BMJ have analyzed data, interpreted and written the final draft of this manuscript. The animal model used in this study was obtained from the Laboratory Animal Center (College of Pharmacy, King Saud University, Saudi Arabia). MAKA has conceived the study and its design and obtained research grants for this study. Moreover, both authors have read and approved the final manuscript.
